# Supplementary material for: A tissue microenvironment analogous to certain tumor microenvironments facilitates HIV persistence
Source: Front Immunol. 2026 Aug 4;17:1862864. doi: 10.3389/fimmu.2026.1862864 (PMC13481462; doi:10.3389/fimmu.2026.1862864)
Supplement: Supplementary file 1 [file DataSheet6.pdf]

Supplementary Table 6: Characteristics of the Persistent versus Transient Reservoir that Reflect Aspects of the Tumor Microenvironment

	**Persistent Reservoir**	**Transient Reservoir**
**Cancer gene scoring spatial distribution**	Localized enrichment of cancer-associated genes in SIV-associated regions, with surrounding collagen/fibrotic-related processes	Broad enrichment of cancer-associated genes with relatively higher enrichment in SIV-associated regions
**Immune infiltration**	↑ immune infiltration in SIV-associated regions	↑↑↑↑↑ immune infiltration in SIV-associated regions
**Gene Pathways**	SIV-positive, high-density areas: energetic metabolic pathways<br><br>SIV-positive, low-density areas: stromal activation, EMT, TGFβ, TNF, and CAF-associated pathways	SIV-positive, high-density areas: Activation of $CD8^+$ T cell, antigenic, and nucleic/amino acid metabolic pathways
**Cell Interactions**	Tregs receive signals from various, notably innate, immune cells in the microenvironment	Tregs send signals to various adaptive immune cells in the microenvironment<br><br>Contact interactions of SIV-associated Th17 and $CD8^+$ T cells
**Top Unique Host transcriptional markers**	KRT8<br>EPCAM<br>MLEC	KRT18<br>TSPAN8<br>SASH3
